# Would a complete electrophysiological study allow us to make a correct diagnosis? Case report

**DOI:** 10.1186/s43044-023-00362-5

**Published:** 2023-05-01

**Authors:** Elibet Chávez-González, Raimundo Carmona-Puerta, Fernando Rodríguez-González, Juan Miguel Cruz-Elizundia, Cynthia Torres-Acosta

**Affiliations:** 1 Electrophysiology Department, Cardiocentro Ernesto Che Guevara, Calle Cuba 610, 50200 Santa Clara, Villa Clara Cuba; 2 Emergency Cardiology Service, Gustavo Aldereguía Lima Hospital, Cienfuegos, Cuba

**Keywords:** Wolff-Parkinson-white, Epicardial accesory pathway, Radiofrequency ablation, Oblique accessory pathway, Case report

## Abstract

**Background:**

Oblique course of some left accessory pathways is rare An incomplete electrophysiological study may confuse us between an oblique accessory pathway or the presence of two accessory pathways. The proximity of all atrial and ventricular electrograms, at each pole of the catheter, within the coronary sinus may be a novel finding.

**Case presentation:**

A 68-year-old woman patient presented arrhythmias with hypotension requiring electrical cardioversion. Her electrocardiogram (ECG) was interpreted as atrial fibrillation by accessory pathway. We performed with the protocol of ablation stablished in our laboratory: two punctures on the right femoral vein with placement of introducers (8F and 7F) by Seldigner technique and one puncture on the left femoral vein (7F). The study was performed with BIOTRONIK technology (Multicath study catheter), a non-deflectable 7F quadripolar catheter with 2 mm tip electrode to record the His electrogram, a non-deflectable decapolar catheter with 5 pairs of coronary sinus (CS) electrodes. Accessory pathway mapping was performed in right and left cavities and within the CS. All electrograms into CS showed short AV from proximal to distal CS. Finally, ablation of two accessory pathway recordings was achieved at two distant epicardial points within the CS.

**Conclusions:**

Ablation at two distant sites, one on the ventricular side and the other on the mitral annulus, suggests the presence of an oblique accessory pathway and at the same time the differential diagnosis of the presence of two accessory pathways. In our point of view according the above, we consider this is a very rare case of oblique AP with epicardial trajectory. The sequence of electrograms (in this case) along the CS has not been seen before in the literature reviewed. It is important, regardless of the urgency, to follow diagnostic and therapeutic protocols in invasive electrophysiology.

## Background

Oblique course of some left accessory pathways (APs) is rare and has been verified by activation of their atrial and ventricular insertion after reversal of the direction of ventricular and atrial wavefronts revealing the oblique course [1]. Electrograms after atrial or ventricular pacing could demonstrate far distant from the ventricular and atrial insertion zones, e.g., ventricular insertion near the ostium of the Coronary Sinus (CS) and atrial insertion on the posterolateral or lateral wall on the Mitral annulus (shorter AV in the medial or lateral region of the CS recordings). When ablation attempts by very expertise operators fail either by transseptal or retrograde transaortic route we should think about epicardial AP and it is still rarer [2]. In 3187 patients taken to ablation, only 5 of them (0.2%) presented epicardial AP [3, 4]. The sequence of electrograms within the coronary sinus is well described in the literature for oblique accessory pathways [1–5]. We describe a new finding that may suggest an oblique AP with two distant epicardial ablation points through the CS.

## Case presentation

**Fig. 1:**
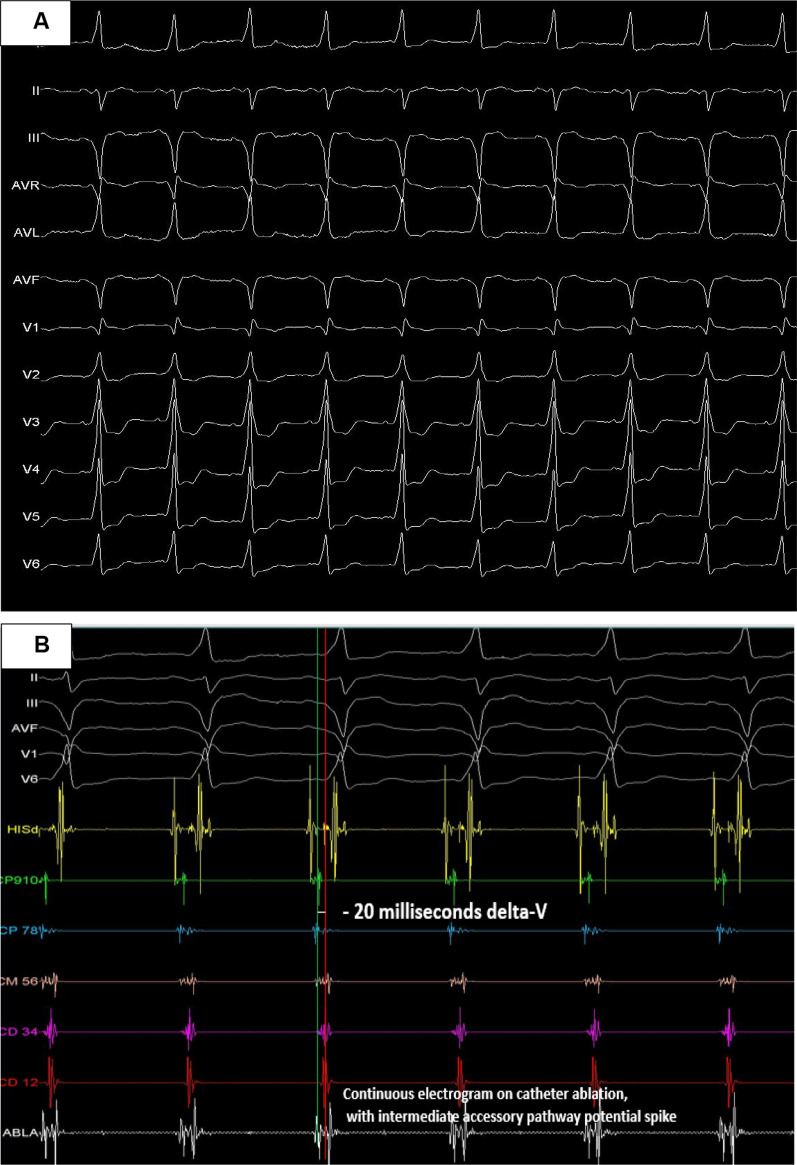
**A** 12-lead electrocardiogram showing the accessory pathway. **B** electrograms recording on His, into the coronary sinus with all short AV and recording of catheter ablation

A 68-year-old woman patient presented arrhythmias with hypotension requiring electrical cardioversion. Her electrocardiogram (ECG) was interpreted as atrial fibrillation (AF) by AP. She was brought urgently to our electrophysiology laboratory, where we are trained for electroanatomical mapping but it is not available. The ECG suggested the presence of posterior or right postero-septal AP (Fig. 1A);

In this case, with recurrent AF by AP and hypotension we did not decide to perform the electrophysiological study to determine the AP insertion sites due to the patient's clinical condition. Cardiologists who was in charge of the patient requested our intervention due to despite the group I-C antiarrhythmic drugs treatment (at recommended doses) the patient continued suffering AF by AP. Once in the electrophysiology laboratory secondary to catheters movement into the heart AF by AP began with hypotension and it was necessary to perform electrical cardioversión (2 Joules per kilogram of weight was calculated, discharge was synchronized with QRS of the electrocardiogram). A deep sedation was performed with midazolam (10 mg ampules in 2 ml), initial dose of 0.5 mg. A single shock was required to recover sinus rhythm.

Considering the above we proceeded with the protocol of ablation stablished in our laboratory: two punctures on the right femoral vein with placement of introducers (8F and 7F) by Seldigner technique and one puncture on the left femoral vein (7F). The study was performed with BIOTRONIK technology (Multicath study catheter), a non-deflectable 7F quadripolar catheter with 2 mm tip electrode to record the His electrogram, a non-deflectable decapolar catheter with 5 pairs of CS electrodes. All electrograms into CS showed short AV from proximal to distal CS (Fig. 1B).

Using a BIOTRONIK ablation catheter (Alcath gold fullcircle) through the 8F introducer. Delta wave in V1 showed negative suggesting the septal region accessory pathway.

It seemed ventricular electrogram CS proximal (CP 9–10) was earliest during sinus rhythm in Fig. 1B. It is important to validate the electrogram component between atrium and ventricle. Nevertheless, AP mapping on the right posterior septal region showed no accessory pathway recordings. That is why we decided to perform anterograde mapping of the AP into the left cavities, using a St Jude deflectable sheath (Agilis 8.5 F) and by transseptal puncture we accessed to the left atrium. Mapping with the ablation catheter of the entire posteroseptal, posterior, posterolateral and lateral region of the mitral annulus, we never saw an AV shorter than those observed in the CS; the shorter AV into left chambers was observed on posteriorseptal región (delta-V of 5 ms), then we decided to apply radiofrecuency (RF) on there (temperature of 60 degrees, 55 watts and 60 s of application); finally it was not successful.

**Fig. 2:**
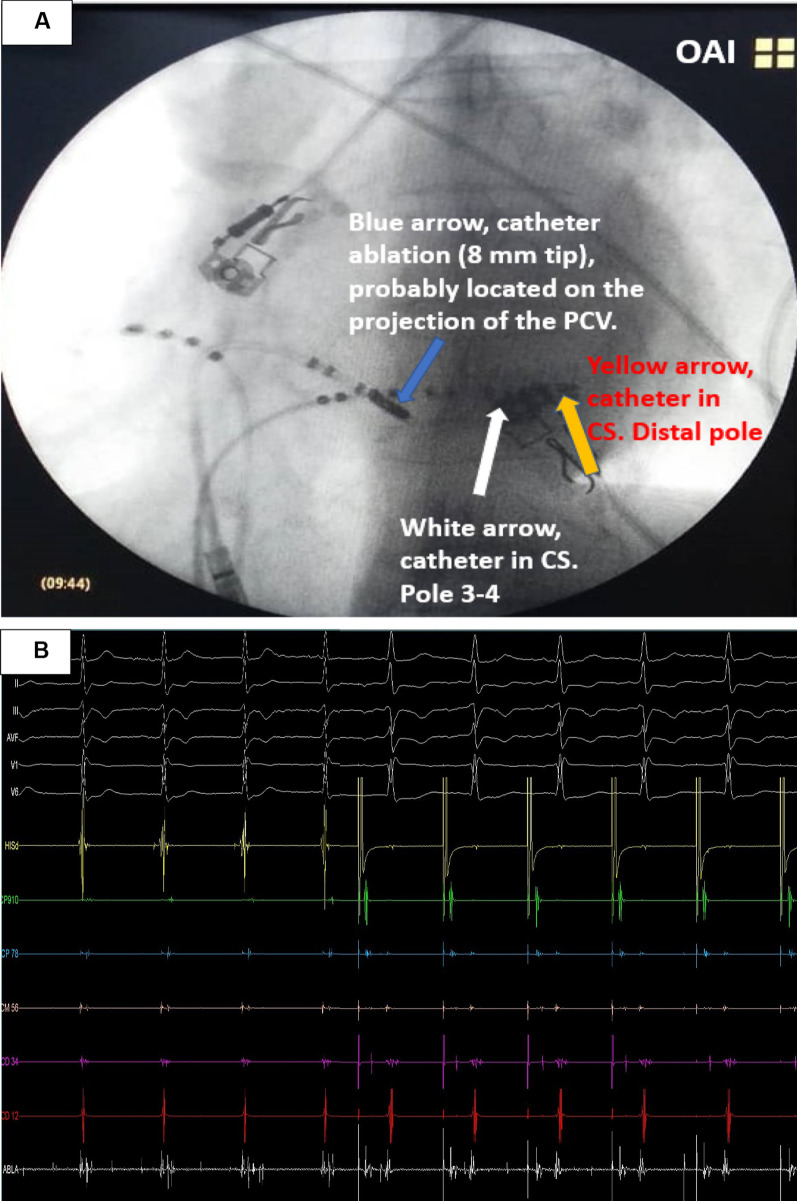
**A** Location of the catheter ablation, probably in the left posterior vein where the radiofrequency was applied. **B** Beginning of junctional rhythm and start of right atrial pacing

We returned to the right cavities decided to map in sinus rhythm the entire CS from its proximal to the most distal portion, observing AP recordings with very short AV along the CS, the shortest delta-V was − 20 ms, always on proximal CS (CS 9–10), Fig. 1B. Nevertheless, the AP electrogram was not found nearby of it. Finally we decided to map into the CS finding the a continuous electrogram on ablation catheter, with intermediate accessory pathway potential spike into of the left posterior coronary vein (PCV) considering its probable radiological projection and here was applied RF with 50 watts and 50 Celsius degrees on the PCV anatomical region (Fig. 2A). During the first seconds of radiofrequency application the patient referred intense pain, so we decided a deep sedation with midazolam newly, this time 1 mg to achieve deep sedation. A trained specialist was in charge of the patient's airway management. This allowed us to gradually increase the temperature for ablation and fortunately there were no impedance increases in the ablation channel. It allowed us to continue and end the procedure.

After we decided to increase 55 watts and 60 Celsius degrees; a junctional rhythm started and we moved the His recording catheter to the right atrium and started to pace with a higher cycle length than the junctional rhythm (Fig. 2B) after 60 consecutive seconds of RF the AP was ablated on the ventricular site. We could recognize the accessory pathway was ablated because during pacing from the right atrium a normal PR segment can be observed on the surface electrocardiogram, without the presence of a delta wave (Fig. 2B). If accessory pathway were still present, pacing from the atrium at a basic cycle higher than junctional rhythm would reveal the presence of the accessory pathway (delta wave on electrocardiogram).

The radiological projection suggests we were probably in the posterior coronary vein; however, a venography should have been performed to identify that site; there is a anatomical variation of the CS ostium. Some of the patients showed large ostium with trumpet-shape. It is still unclear the shortest delta-V was located at left posterior vein without CS venography or Geometry with 3D mapping system. In our laboratory we have not a 3D system, we should have performed CS venography and it was not performed. It was a limitation to diagnosis the anatomical site of ablation.

**Fig. 3:**
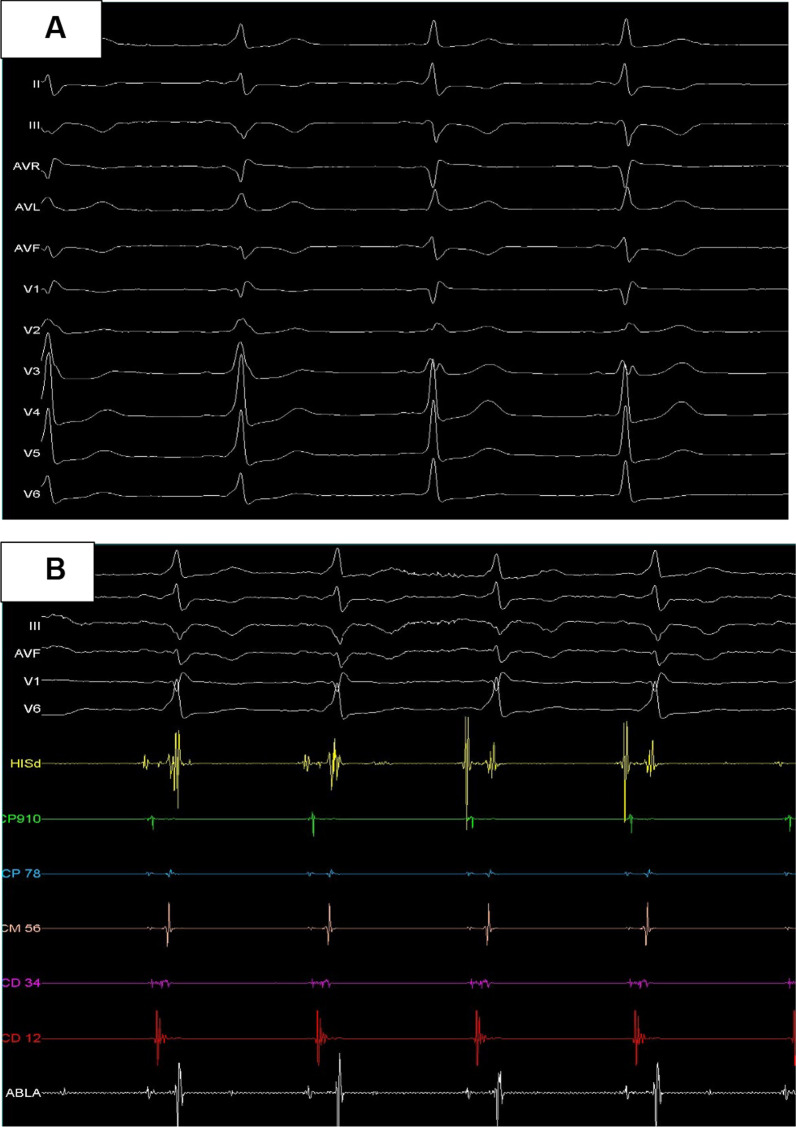

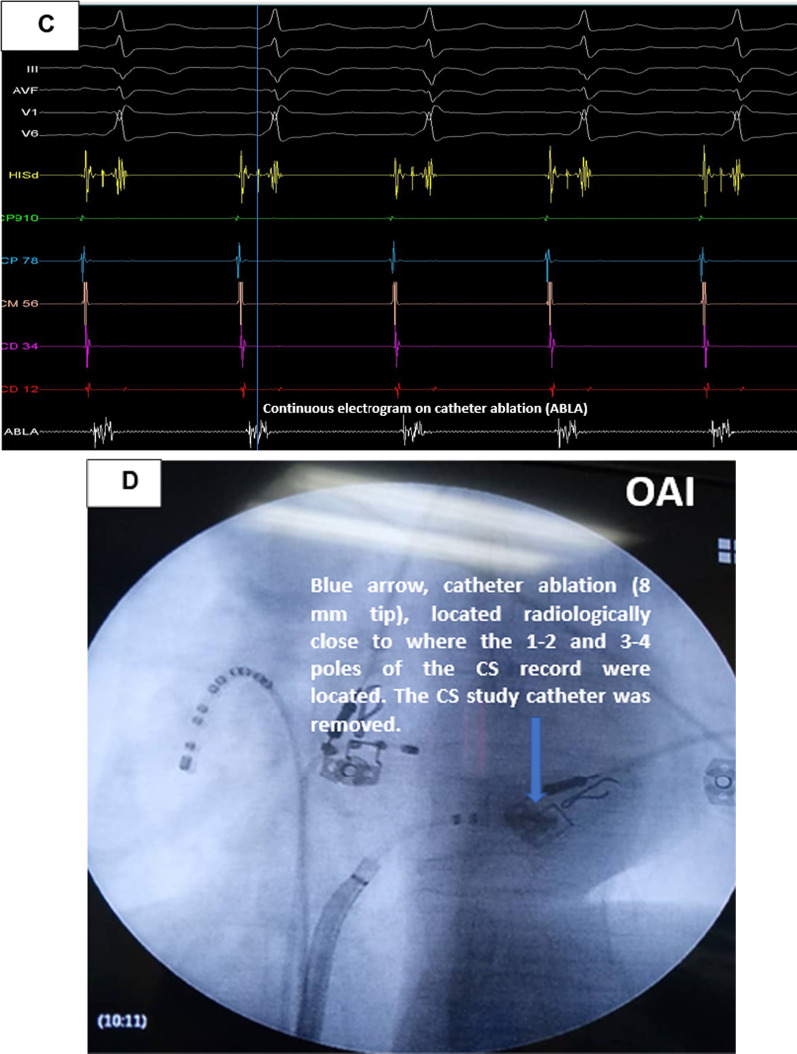
**A** Electrocardiographic pattern of accessory pathway modified after the first ablation site. **B** Coronary sinus electrograms after ablation of the first site, poles 1–2 and 3–4 remain with short AV. **C** Continuous electrogram in the ablation recording showing the AP potential, corresponding to the second ablation site. **D** Ablation catheter tip in the posterolateral area, it was the second ablation site

According to our laboratory protocols, we waited ten minutes after ablation and administered adenosine, 12 mg intravenously for verification of AP ablation. After the administration of adenosine an electrocardiographic pattern reappears with the presence of delta wave (Fig. 3A); however, compared to the initial ECG, a small R wave appears in DII and CS recording has been modified with shorter AV on CS 1–2 and 3–4 (Fig. 3B). Upon observing a new site where the CS 3–4 accessory pathway would be passing through, we decided to stimulate from that point of the atrium (CS 3–4) and observed maximum pre-excitation. We also stimulated from 9–10 CS and there was no change in the magnitude of the surface electrocardiogram delta wave. When pacing from the right ventricle, the first retrograde atrium was observed at CS 3–4. Considering that initially left intracavitary mapping had been performed, we decided to go directly through the CS.

**Fig. 4:**
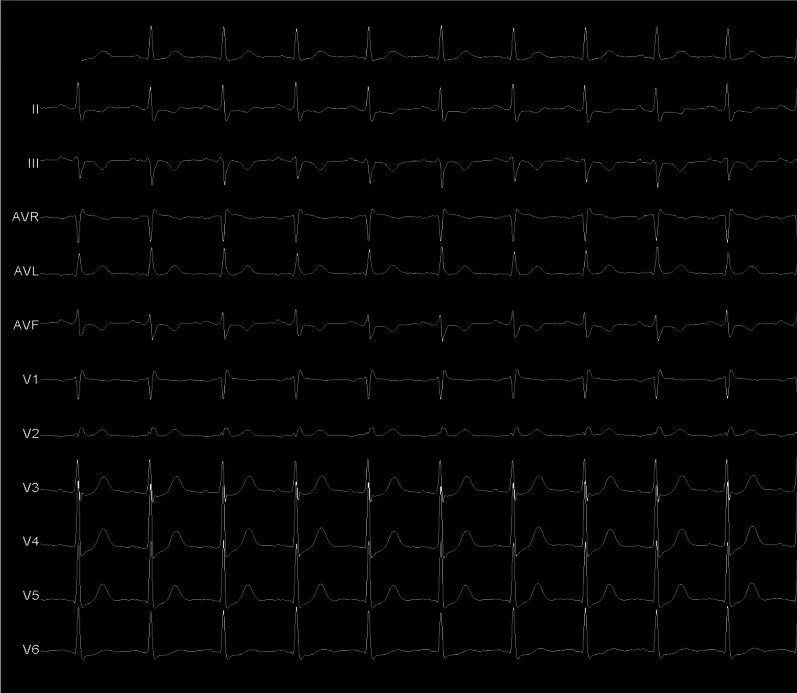
Final electrocardiogram after ablating two anatomical points, there is no evidence of accessory pathway and there is normal PR

Mapping within CS with ablation catheter up to anatomical and fluoroscopic region where the CS catheter recording 3–4 was located (it was removed) (Fig. 3C). Successful ablation was achieved at this second point, on the mitral annulus. After waiting 10 min and administration of adenosine, ablation was successful at two distant epicardial points and the ECG was in sinus rhythm with normal PR (Fig. 4).

## Discussion

In this case, with recurrent AF by AP and hypotension we did not decide to perform the electrophysiological study to determine the AP insertion sites due to the patient's clinical condition. To achieve the ablation site on hidden accessory pathways ventricular pacing is needed or mapping during tachycardia is required. However, it is preferable to perform mapping and radiofrequency ablation during sinus rhythm for manifest pathways; ablation during tachycardia may be hindered by poor catheter stability. If AP is manifested a continuous intracavitary electrogram in the bipolar channel of the ablation catheter could be observed, ventricular electrogram is ahead of the delta wave onset on the surface electrocardiogram [6, 7]. Our clinical case presented with a manifest accessory pathway that is why we proceeded to perform the ablation in sinus rhythm. However, it is necessary to perform an atrial and ventricular pacing protocol in the presence of accessory pathways with atypical electrophysiological manifestations [8]. Finally, it was the protocol that we performed to verify the insertion site of the accessory pathway that was manifested at the second moment with continuous electrogram in 3–4 CS.

After ablation at two distant epicardial sites within the CS, it may be difficult to define between two diagnosis: 1. oblique accessory pathway or 2. the presence of two accessory pathways in this patient.

There are two major limitations do not allow us to reach the right diagnosis: 1. We did not perform the electrophysiological study with pacing from the ventricle and atrium and we could not determine the probable existence of oblique atrial and ventricular insertion sites. 2. CS venography was not performed to identify the anatomical site of the first ablation site.

Despite these limitations we consider this patient is an interesting case report given the unusual behavior of the electrograms within the CS with short AV. It have not found reported in the literature.

APs in sinus rhythm show an earlier ventricular electrogram in one of the CS recordings so that AV distances are different [2, 5]. In our patient the CS recordings all the AV were very short and similar so we decided to map the best AP electrogram recording with negative delta-V in the ablation recording to apply RF. Once an epicardial point is located in the PCV transient abolition of AP conduction is achieved after RF.

Accessory connections from CS to the ventricle produce epicardial posteroseptal or left posterior AP The presence of APs in CS result from a connection between a CS myocardial coat extension along the middle cardiac vein or the PCV and the ventricle [3].

In this case upon achieving transient ablation at the probable anatomical site of the PCV and AP reappears with evident recording in CS 3–4 (Fig. 3B) to finally achieve ablation at that posterolateral epicardial point which leads us to think about an epicardial oblique AP. The transient abolition of AP may explain the failure to ablate AP by RF application on the ventricular side of the mitral annulus. The initial RF application on the ventricular insertion with transient abolition of AP and the final success with RF application on atrial insertion has been the end point to demonstrate the presences of oblique AP [2].

## Conclusions

Having performed the complete electrophysiological study plus CS venography could have helped us to make a correct diagnosis as we have already stated earlier in the discussion. Nevertheless, the electrograms recordings along the entire CS trajectory and upon achieving definitive abolition of AP pattern by ablating at two distant points (ventricular and on the mitral annulus) it leads us to think about an oblique AP presence; in addition, ablation was achieved epicardial intra CS. In our point of view according the above, we consider this is a very rare case of oblique AP with epicardial trajectory. The sequence of electrograms (in this case) along the CS has not been seen before in the literature reviewed.

## Data Availability

No datasets were generated or analyzed during the current study.

## Abbreviations

AF: Atrial fibrillation
APs: Accessory pathway(s)
AV: Atrio-ventricular
CS: Coronary sinus
ECG: Electrocardiogram
PCV: Posterior coronary vein
PR: PR segment of the electrocardiogram
